# Association between Genetic Polymorphism of *SCN1A*, *GABRA1* and *ABCB1* and Drug Responsiveness in Vietnamese Epileptic Children

**DOI:** 10.3390/medicina60040637

**Published:** 2024-04-16

**Authors:** Hai Xuan Tang, Muoi Dang Ho, Nhung Phuong Vu, Hung Vu Cao, Vinh Anh Ngo, Van Thi Nguyen, Thuan Duc Nguyen, Ton Dang Nguyen

**Affiliations:** 1 Nghe An Obstetrics and Pediatrics Hospital, 19 Ton That Tung, Vinh 460000, Nghe An, Vietnam; bstangxuanhai@gmail.com (H.X.T.); dr.dangmuoi@gmail.com (M.D.H.); 2 Institute of Genome Research, Vietnam Academy of Science and Technology, 18 Hoang Quoc Viet, Cau Giay 100000, Hanoi, Vietnam; nhungvp@igr.ac.vn; 3 Vietnam National Children’s Hospital, 18/879 La Thanh, Dong Da 100000, Hanoi, Vietnam; hungcv@nch.gov.vn (H.V.C.); drngovinh@gmail.com (V.A.N.); drvannguyen90@gmail.com (V.T.N.); 4 Department of Neurology, Military Hospital 103, Vietnam Military Medical University, 261 Phung Hung, Ha Dong 100000, Hanoi, Vietnam; nguyenducthuan@vmmu.edu.vn

**Keywords:** epilepsy, drug-resistant epilepsy, SCN1A, GABRA1, ABCB1

## Abstract

*Background and Objectives:* Drug resistant epilepsy (DRE) is a major hurdle in epilepsy, which hinders clinical care, patients’ management and treatment outcomes. DRE may partially result from genetic variants that alter proteins responsible for drug targets and drug transporters in the brain. We aimed to examine the relationship between *SCN1A*, *GABRA1* and *ABCB1* polymorphism and drug response in epilepsy children in Vietnam. *Materials and Methods*: In total, 213 children diagnosed with epilepsy were recruited in this study (101 were drug responsive and 112 were drug resistant). Sanger sequencing had been performed in order to detect six single nucleotide polymorphisms (SNPs) belonging to *SCN1A* (rs2298771, rs3812718, rs10188577), *GABRA1* (rs2279020) and *ABCB1* (rs1128503, rs1045642) in study group. The link between SNPs and drug response status was examined by the Chi-squared test or the Fisher’s exact test. *Results*: Among six investigated SNPs, two SNPs showed significant difference between the responsive and the resistant group. Among those, heterozygous genotype of *SCN1A* rs2298771 (AG) were at higher frequency in the resistant patients compared with responsive patients, playing as risk factor of refractory epilepsy. Conversely, the heterozygous genotype of *SCN1A* rs3812718 (CT) was significantly lower in the resistant compared with the responsive group. No significant association was found between the remaining four SNPs and drug response. *Conclusions*: Our study demonstrated a significant association between the *SCN1A* genetic polymorphism which increased risk of drug-resistant epilepsy in Vietnamese epileptic children. This important finding further supports the underlying molecular mechanisms of *SCN1A* genetic variants in the pathogenesis of drug-resistant epilepsy in children.

## 1. Introduction

Epilepsy is the most common neurological disorder in children, which affects 41–187/100,000 live births, and a higher incidence was reported in the underdeveloped countries [1]. Drug-resistant epilepsy (DRE) remains a considerable obstacle in the management of epilepsy, affecting approximately 30% of patients who do not achieve seizure control despite the availability of over 20 anti-epilepsy drugs (AEDs) [2]. DRE occurs when two appropriate AEDs are prescribed adequately, either as a monotherapy or combination therapy but fail to control patients’ seizures. Drug responses in epilepsy patients are highly variable and unpredictable, which depends on multiple factors, such as epilepsy types, AEDs target, AEDs pharmacokinetics and genetics. There are two mostly accepted hypotheses of factors underlying DRE mechanisms, including target hypothesis and transporter hypothesis [3]. The voltage-gated sodium channels are responsible for membrane transportation of Na^+^ ions, which is required for generating excitability of neuronal cells [4]. Gamma-aminobutyric acid (GABA) receptors reduce neuronal excitability via inhibiting transmission between nerve cells [5]. Both sodium channels and GABA receptors are important molecular targets of variable AEDs such as carbamazepine, ox-carbamazepine, phenytoin, lamotrigine and valproic acid [6]. The ATP binding cassette (ABC) transporters play an important role in maintaining brain homeostasis via extruding unwanted compounds from blood [7]. For the target hypothesis, it was proposed that malformations in AED targets (voltage-gated ion channels and neurotransmitter receptors) ultimately lead to attenuated AED sensitivity and observed refractory phenomenon [8]. For the transporter hypothesis, it was assumed that overexpression of efflux transporters at the blood–brain barrier, which impairs adequate penetrance AEDs into the epileptic region despite the sufficient drug level in patient’s plasma, could be one mechanism of the refractory phenomenon [9]. From a genetic perspective, variants that potentially alter the expression or function of drug targets and transporter proteins may also contribute to the DRE pathogenesis by altering the excitability and connectivity of seizure networks [10]. Mechanistically, from a pharmacogenetics perspective, variable drug responses are substantially attributed to genetic variants through alteration of pharmacokinetics and pharmacodynamics of AEDs. This is a promising avenue to optimize pharmacotherapy and ultimately overcome DRE [11]. From the past 20 years, association between single nucleotide polymorphisms (SNPs) in the number of genes encoding AEDs targets and transporters with DRE have been investigated, in which SNPs belonging to GABRA1, SCN1A and ABCB1 were extensively studied in numerous ethnic groups [12,13,14,15,16]. However, to the best of the authors’ knowledge, the exact mechanism involved in AED resistance remains unknown. Given that much controversial evidence has been drawn from pharmacogene variation and DRE association, large-scale studies involving diverse population are amenable to further solidify the hypotheses of the DRE mechanism. In the near future, a solid investigation would support the development of individualized treatment strategies based on personal genetic profiles and finally overcome DRE. 

In the present study, we aim to find the association of SNPs in three genes including *SCN1A*, *GABRA1* and *ABCB1* in Vietnamese children affected by epilepsy. This is valuable evidence of the genetic risk factor of pharmacoresistant epilepsy, which is firstly investigated in Vietnam. 

## 2. Method

### 2.1. Subject Collection and Clinical Classification

The study aim was explained to all participants and their sponsors before sample collection. All volunteers read and signed the informed consent forms, which provide information for making decisions of the enrolled subjects. This work obtained ethical approval from the Ethics Committee of Vietnam National Children’s Hospital (No: VN01037/IRB00011976/FWA00028418). 

In total, 213 children were diagnosed with epilepsy by neurologists from Nghe An Obstetrics and Pediatrics Hospital, Vietnam National Children’s Hospital and Military Hospital 103. Among those, 101 were drug-responsive and 112 were drug-resistant. According to the International League Against Epilepsy, drug-resistant epilepsy was defined as the “failure of an adequate trial of two tolerated and appropriately chosen antiepileptic drug-AED schedules (whether as monotherapies or in combination) to achieve seizure freedom”, and drug-responsive epilepsy was defined as “with current AED, the patient has been seizure free for a minimum of three times the longest pretreatment inter seizure interval, or 12 months, whichever is longer”.

### 2.2. Inclusion and Exclusion Criteria

Inclusion criteria: Children diagnosed with epilepsy (both responsive and resistant according to the ILAE definition), age from 1–15 years old and legal guardians agreed to participate in this study. 

Exclusion criteria: Children diagnosed with epilepsy but did not follow treatment adherence, have a history of brain injury, brain tumor and/or infection causing acquired epilepsy, medical records were not available and legal guardians refused to participate in this study. 

### 2.3. DNA Extraction and Sanger Sequencing

For all participants, 2 mL of peripheral blood was collected in an EDTA-containing tube and stored at −20 °C. Genomic DNA was subsequently extracted from 200 μL of total blood by the Exgene Blood^TM^ SV kit following the manufacturer’s protocol (GeneAll, Seoul, Republic of Korea). Afterward, the concentration of extracted DNA was determined by a Qubit dsDNA BR Assay kit (ThermoFisher Scientific, Waltham, MA, USA).

Primers were designed specifically for selected SNPs of *SCN1A*, *GABRA1* and *ABCB1* (Table 1). All primers were synthesized and provided by Phusa BioChem, Can Tho, Vietnam. For *SCN1A* (rs2298771, rs10188577), *GABRA1* (rs2279020) and *ABCB1* (rs1128503, rs2032582, rs1045642), each PCR reaction was 20 µL in total, including 10 ng genomic DNA, 10 µL of Dream Taq Master Mix (ThermoFisher Scientific), 1 µL of each primer (10 pmole/µL) and 7 µL of deionized water (ThermoFisher Scientific). The thermo-cycle was as follows: denaturation at 95 °C for 5 min, followed by 40 cycles of 95 °C for 30 s, 58 °C (rs2298771, rs10188577, rs1128503, rs2032582, rs1045642)/60 °C (rs2279020) for 30 s, 72 °C for 30 sec and a final extension at 72 °C for 10 min. For *SCN1A* (rs3812718), PCR reaction was performed with volume of 20 µL in total, including 10 ng genomic DNA, 10 µL of NEB Master Mix (NewEngland Biolab, Ipswich, MA, USA), 1 µL of each primer (10 pmole/μL), 0.5 µL of MgCl_2_ and 6.5 μL of deionized water (ThermoFisher Scientific). The thermo-cycle was as follows: denaturation at 95 °C for 5 min, followed by 40 cycles of 95 °C for 30 s, 52 °C for 15 s, 72 °C for 30 s and a final extension at 72 °C for 10 min. 

All amplicons were later purified using Multiscreen Filter Plate (Merck Millipore, Burlington, MA, USA) and subsequently sequenced on an ABI genetic analyzer using the BigDye^TM^ Terminator v3.1 Cycle Sequencing Kit (ThermoFisher Scientific). 

### 2.4. Data Analysis

Sanger sequencing data were analyzed by Bioedit software ver 7.2 according to the Reference sequence of genes including *SCN1A* (NG_011906.1), *GABRA1* (NG_011548.1) and *ABCB1* (NG_011513.1). All reference sequences were referred from NCBI GeneBank.

Statistical analyses were performed by R packages. The Hardy–Weinberg equilibrium and the difference between categorical variables were examined by Chi-squared and Fisher’s exact tests. An independent sample T test was used to analyze the association between continuous variables. A logistic regression model was applied to evaluate the effect of multiple factors on drug response. A *p* value < 0.05 was considered as statistical significance. The odds ratio (OR) was calculated for a *p* value < 0.05. 

## 3. Results

### 3.1. General Features of Enrolled Participants

In this study, a total of 213 children diagnosed with epilepsy were recruited. Among those, 101 patients were responsive (55 male and 46 female), and 112 patients (57 male and 55 female) were resistant. No association was found between gender and DRE risk in the study groups. The mean age of the DRE group was 48.14 ± 4.16 months versus 69.17 ± 4.38 months in responsive group. In both the resistant and control groups, most of the children were affected by local epilepsy (77/101 in the responsive group and 74/112 in the DRE group), and the combined generalized and focal epilepsy is less frequent. Furthermore, abnormal MRI-EEG, history of febrile seizure and infantile spasms were also significantly prevalent in the DRE group compared with the responsive group (*p* < 0.05). All preclinical and clinical characteristics of epileptic children are presented in Table 2. 

### 3.2. Frequency of SCN1A, GABRA1 and ABCB1 Genotypes and Alleles in Study Group

All six SNPs of *SCN1A*, *GABRA1* and *ABCB1* showed no deviation from the Hardy–Weinberg equilibrium (HWE *p* value > 0.05, Table S1).

Among the six variants assessed, only two of *SCN1A* showed a significant difference between the responsive group and resistant group. For rs2298771, compared to the reference genotype, the proportion of the heterozygous genotype GA in the DRE group was significantly higher than that in the control group (*p* = 0.037; OR = 2.019). In addition, the recessive model (AA/GG + GA) made up 71.4% and 83.16% in the resistant group and responsive group, respectively. This difference reached the statistical threshold (*p* = 0.042, OR = 0.5). For rs3812718, compared to the CC genotype, the heterozygous genotype CT was detected at a significantly higher percentage in the responsive group (52.5%) compared with that in the resistant group (34.8%) (*p* = 0.002; OR = 0.304). Additionally, the dominant model (CT + TT/CC) accounted for 89.1% in the responsive group, which was significantly higher than that in the resistant group (*p* = 0.011, OR = 0.389).

No difference was found in genotype frequencies of the remaining SNPs, including *GABRA1* (rs2279020) and *ABCB1* (rs1128503, rs1045642). Similarly, allele frequencies of all studied SNPs were comparable among the DRE group and control group (*p* > 0.05). All studied SNP genotype and allele frequencies are presented in Table 3 and Table 4, respectively.

### 3.3. Association of Haplotypes with Drug Response in Study Group

In total, 16 haplotypes *SCN1A* and 4 haplotypes of *ABCB1* were composed from three SNPs of *SCN1A* (rs2298771, rs3812718, rs10188577) and two SNPs of *ABCB1* (rs1128503, rs1045642). However, among these 20 haplotypes, statistical analysis showed no difference between DRE and the control group (*p* > 0.05) (Table 5).

### 3.4. Logistic Regression Analysis of Factors Affecting Epileptic Drug Response

We further analyze the correlation between two SNPs of *SCN1A* (rs2298771, rs3812718) and DRE by the adjusted logistic regression model. However, there is no statistically significant relationship between the two SNPs and the drug response in the two groups. Patients with a history of intellectual disability and febrile seizure were at a higher risk of being resistant, and the data reached statistical significant (*p* < 0.01) (Table 6).

## 4. Discussion

Drug-resistant epilepsy affects approximately one-third of epilepsy patients who do not achieve seizure freedom regardless of an adequate drug prescription [17]. Prolonged and uncontrolled seizures are the cause of serious complications including cognitive impairments, depression, injury and even sudden death. Early identification of individuals at risk of DRE is crucial. This helps to mitigate the burden of this disease and prevents using unsuitable drugs as well as facilitating the selection of alternative drug therapies.

Voltage-gated sodium channels play an important role in proper neurological function, in which the *SCN1A* gene encodes a sodium channel called NaV1.1. This is a transmembrane protein in the brain which is responsible for allowing sodium ions to cross the membrane, finally managing the interaction between nerve cells through neurotransmitters. Therefore, these channels are the target of many first-line AEDs that have been widely used. The rs2298771 G > A (p.Thr1067Ala), rs3812718 (IVS5N+5 C > T) and rs10188577 A > G are common polymorphisms of the *SCN1A* gene, which were extensively studied in various populations regarding their relationship with drug responses [18,19,20,21,22]. For rs2298771 G > A (p.Thr1067Ala), this is a variant in the coding region of the *SCN1A* gene. We found that the heterozygous genotype GA is a risk factor of DRE. The recessive model also showed that carriers of the G allele (GG + GA) were at higher risk of being drug-resistant. There were limited individuals with the GG genotype in both studies groups, therefore no statistical significance was found regarding the homozygous GG genotype among the two groups. Our data are consistent with several investigations where the GA genotype and G allele have been demonstrated as predictors of refractory epilepsy [23,24]. In a longitudinal clinical follow-up from 3 months to 12 months with Carbamazepine administered as a monotherapy, the percentage of patients carrying the AA genotype (rs2298771 G > A) and being seizure-free was significantly higher than those with the AG + GG genotype [25]. It has been suggested that the replacement of threonine by alanine can influence the conformational and functional properties of sodium channels, leading to differential responses to sodium channel blockers [24]. For rs3812718 C > T (IVS5N+5C > T), this SNP is located in the upstream sequence of exon 5 and can alter the proportions of adult and neonatal transcripts of *SCN1A* through alternative splicing [26]. Indeed, previous studies found that the allele T leads to the disruption of the splice donor site of exon 5 and consequently inhibits the expression of exon 5N (neonatal transcript) but increases the expression of exon 5A (adult transcript) [26,27]. The alternative transcript level of exon 5N in the human brain tissue was highest in the samples with the CC genotype, which was consistent with that found in the minigene system. This evidence proved that exon 5N expression was directly affected by the *SCN1A* genotype [26]. Mechanistically, the replacement of C by T disrupts the conserved consensus-site sequence and theoretically creates a weaker 5′ splice site [28]. Our study revealed that the CT genotype was more prevalent in the well-responsive patients than in the DRE patients. As expected, the dominant model (CC + CT) was also found to be a protective factor against refractory epilepsy. Remarkably, a cross-sectional study on Japanese epilepsy patients has demonstrated that the variant rs3812718 can impact the resistant status to carbamazepine and co-administered valproic acid [29]. Additionally, in Chinese epilepsy patients, individuals with the homozygous genotype TT of rs3812718 were less sensitive to the sodium channel blocking drugs and at higher risk to developing drug resistance [30]. Mechanistically, the elevated expression of exon 5A might substantially contribute to the more sensitivity with AEDs in epileptic children. For rs10188577 A > G, this is also a common SNP of the intron region and is considered as a critical regulator of *SCN1A* expression via affecting the transcription factor or methylation [22,31]. The study of Feng et al. found that *SCN1A* rs10188577 was linked to valproic acid resistance in Chinese children affected by generalized epilepsy [32]. There was only moderate significant difference in epilepsy in Caucasians who were treated with sodium channel blockers (phenytoin, carbamazepine, topiramate and valproic acid), in which heterozygous carriers (AG) were found with a higher frequency in the drug-resistant group [31]. Moreover, other research did not support this correlation [33]. To date, the exact effect of *SCN1A* variants on DRE pathogenesis remains unknown, the difference in ethnicity, epilepsy type of enrolled patients and concomitant medications perhaps contribute to the inconsistent results.

GABA is the major inhibitory neurotransmitter that regulates neuronal excitability and network interactions in the cerebral cortex. This inhibitor plays a significant role in behavior, cognition and stress responses of individuals. It acts via three receptor classes: the ionotropic GABAA and GABAC receptors (ligand-gated ion channels, opening chloride channels) and the metabotropic GABAB receptors (G protein-coupled receptors, regulating potassium and calcium channels). Similar to SCN1A, GABRA1 was considered as one of AEDs’ main targets through maintaining the homeostasis over brain excitation [34]. The SNP rs2279020 of *GABRA1* is an intronic polymorphism, which possibly alters the structural properties of the mature protein through influencing the mRNA splicing [35]. Various studies have searched for associations between *GABRA1* SNPs and DRE including rs2279020; however, data released were still controversial. For example, in Jordan, patients carrying the G allele (GG + GA) tend to develop AED resistance [36]. Similarly, in Indian patients, both the GG genotype and G allele were demonstrated as risk factors of multiple drug resistance [35]. On the contrary, there was no association of *GABRA1* rs2279020 with predisposition of AEDs resistance in Asian and Arabic populations [37,38]. The *ABCB1* gene encodes P-glycoprotein and the efflux transporter that modulates antiepileptic drug pharmacokinetics through the absorption process in the intestine and brain penetrance. It has been widely hypothesized that DRE pathophysiology was caused by P-glycoprotein overexpression [17,39]. Among SNPs of the *ABCB1* gene, biological significance of rs1128503 and rs1045642 has been well studied in multidrug resistance, including AEDs. The SNP rs1045642 T > C is a synonymous variant, in which individuals with the CC genotype showed 2-fold higher P-glycoprotein in the duodenum compared with individuals carrying the TT genotype [40]. These two SNPs are also in a linkage disequilibrium. Many studies have examined the relationship between variants of *ABCB1* (mostly common rs1128503, rs1045642) and refractory epilepsy, but the data are still inconsistent [19,41,42]. In the present study, we failed to detect a correlation between *GABRA1* (rs2279020), *ABCB1* (rs1128503, rs1045642) and DRE, both at the genotype and allele level. Further replication research should be performed in order to confirm this observation. Additionally, in this study, AEDs were not prescribed as monotherapy for epileptic children. The multiple drugs used were possibly a factor affecting the analysis power since not all of them are substrates of P-glycoprotein as well as targeting the GABRA1 receptor.

This study design has several limitations. Firstly, the association between genetic factors and drug response were performed with a relatively limited samples size, which can influence the statistical power. Secondly, the concentration of AEDs in the brain of epileptic children in both groups were unknown. In further studies, data from Magnetic Resonance Spectroscopy based on in vivo models could further estimate the concentration of drug metabolites in the brain, supporting the evidence related to the transporter hypothesis. Thirdly, AED levels in the blood of epileptic children were not measured, and this parameter could vary among individuals, partially depending on the personal pharmacokinetics. Studying the polymorphism of AED metabolism genes is also an important aspect regarding the drug dosage and relative patient response. Furthermore, a genome-wide association study is essential in order to explore the leading SNPs involved in DRE, especially SNPs with low frequency, consequently providing more evidence that aids in the treatment strategies in a personalized manner.

## 5. Conclusions

In present work, we found that rs2298771 (AG genotype) and rs3812718 (CT) of *SCN1A* increased the risk of being vulnerable to drug resistance in Vietnamese epileptic children. There is no significant association between *SCN1A* (rs10188577), *GABRA1* (rs2279020), *ABCB1* (rs1128503, rs1045642) and drug response status according to the current evidence. In the near future, further studies with a larger sample combined with a longitudinal follow-up are required to support the pathogenesis of pharmaco-resistant epilepsy.

## Figures and Tables

**Table 1:** Primers sequences used for PCR and sequencing.

Gene	SNP	Primer Sequence	Expected PCR Product
*SCN1A*	rs2298771-F	TTC TGG CCT TGC TTC TGA GC	498
	rs2298771-R	ATA CCT TCC CAC ACC TAT AG	
	rs3812718-F	GCT CGG AGA ACT CTG AAT G	379
	rs3812718-R	TCT AAT TCC TGA TTT ACC AC	
	rs10188577-F	CAC CAT CAA ACC CAC TCT TG	344
	rs10188577-R	GAA AAC ATT GAG TCA GAG CC	
*GABRA1*	rs2279020-F	TAA GGT GGC TTA TGC AAC AG	362
	rs2279020-R	AAA TGA CCT CTC CCT TTA TC	
*ABCB1*	rs1128503-F	ATC ACC GCA GGG TCT AGC TC	367
	rs1128503-R	TCA CTT CAG TTA CCC ATC TC	
	rs1045642-F	TAA GGG TGT GAT TTG GTT GC	337
	rs1045642-R	GTT TTC AGC TGC TTG ATG G	

Forward primers were used for Sanger sequencing.

**Table 2:** Demographic, preclinical and clinical characteristics of enrolled participants.

Characters	Responsive N = 101 (%)	DRE N = 112 (%)	*p* Value	OR (95% CI)
Age (month, mean ± SD)	69.17 ± 4.38	48.14 ± 4.16	0.001 *	-
Gender			0.36 ^#^	-
Male	55 (54.45)	54 (48.21)		
Female	46 (45.55)	58 (51.79)		
Number of AEDs used (mean ± SD)	1.2 ± 0.69	2.91 ± 1.07	<0.001 *	-
Epilepsy type				
Focal epilepsy	77 (76.23)	74 (66.07)	0.1 ^#^	-
Generalized epilepsy	20 (19.8)	31 (27.68)	0.18 ^#^	-
Combined generalized and focal	4 (3.97)	7 (6.25)	0.45 ^#^	-
Unknown epilepsy	0	0		
Age at onset (month, mean ± SD)	35.84 ± 3.28	11.24 ± 1.12	<0.001 *	-
Duration of epilepsy (minute, mean ± SD)	1.6 ± 1.3	1.8 ± 0.9	0.192 *	-
Frequency of seizure (mean ± SD/month)	5.73 ± 0.55	9.99 ±0.81	<0.001 *	-
History of febrile seizure				
Yes	18 (17.82)	40 (35.71)	0.0039 ^#^	2.56 (1.35–4.85)
No	83 (82.18)	72 (64.29)		
History of infantile spasms				
Yes	3 (2.97)	17 (15.17)	0.006 ^#^	5.85 (1.66–20.59)
No	98 (97.03)	95 (84.83)		
Abnormal MRI				
Yes	15 (14.85)	66 (58.93)	<0.0001 *	8.22 (4.23–15.99)
No	86 (85.15)	46 (41.07)		
Abnormal EEG				
Yes	46 (45.54)	104 (92.85)	<0.0001 *	18.36 (8.1–41.39)
No	65 (54.46)	8 (7.15)		
Intellectual disability				
Yes	37 (33.03)	105 (93.75)	<0.0001 *	0.04 (0.02–0.1)
No	64 (66.97)	8 (6.25)		

N: number of patients; *: independent sample T test; ^#^: Chi square test.

**Table 3:** Distribution of *SCN1A*, *GABRA1* and *ABCB1* genotype frequencies between DRE and responsive controls.

Genotype	Responsive N = 101 (%)	DRE N = 112 (%)	*p*	OR	95% CI
*SCN1A* rs2298771					
AA	84	80		Ref	
AG	16 (15.8)	31 (27.7)	0.037	2.019	1.035–4.068
GG	1	1	0.972 ^*^		
AA/GG + GA	84 (83.16)	80 (71.4)	0.042	0.5	0.26–0.982
GA + AA/GG	100	111	0.941	1.109	0.028–43.654
*SCN1A* rs3812718					
CC	11	27		Ref	
CT	53 (52.5)	39 (34.8)	0.002	0.304	0.129–0.676
TT	37	46	0.102	0.512	0.216–1.152
TT/CC + CT	37 (36.63)	46 (41.07)	0.5	0.83	0.475–1.445
CT + TT/CC	90 (89.1)	85 (75.9)	0.011	0.389	0.174–0.818
*SCN1A* rs10188577					
AA	71	79		Ref	
AG	26	31	0.824	1.07	-
GG	4	2	0.352 ^*^	0.467	-
AA + AG/GG	97	110	0.915	1.031	-
GG + AG/AA	30	33	0.915	0.969	-
*GABRA1* rs2279020					
GG	23	25		Ref	
GA	56	66	0.812	1.084	-
AA	22	21	0.757	0.879	-
GG + GA/AA	79	91	0.883	1.032	-
GA + AA/GG	78	87	0.883	0.968	-
*ABCB1* rs1128503					
TT	45	43		Ref	
TC	41	54	0.279	1.375	-
CC	15	15	0.914	1.046	-
TT + TC/CC	86	97	0.704	0.915	-
TC + CC/TT	56	69	0.704	1.091	-
*ABCB1* rs1045642					
TT	15	10		Ref	
TC	53	55	0.324	1.544	-
CC	33	47	0.1	2.111	-
TT + TC/CC	68	65	0.341	0.806	-
TC + CC/TT	86	102	0.341	1.239	-

N: number of subjects; *: Fisher’s exact test.

**Table 4:** Distribution of *SCN1A*, *GABRA1* and *ABCB1* allele frequencies between DRE and responsive controls.

Allele	Responsive (n = 202)	DRE (n = 224)	*p*
*SCN1A* rs2298771			
A	186	193	0.051
G	16	31	
*SCN1A* rs3812718			
C	75	93	0.354
T	127	131	
*SCN1A* rs10188577			
A	168	189	0.735
G	34	35	
*GABRA1* rs2279020			
G	102	116	0.79
A	100	108	
*ABCB1* rs1128503			
T	131	140	0.614
C	71	84	
*ABCB1* rs1045642			
T	83	75	0.104
C	119	149	

n: number of alleles.

**Table 5:** Comparison of haplotype frequencies composed of *SCN1A* and *ABCB1* in study group.

Analysis	Responsive (%) n = 202	DRE (%) n = 224	*p*
*SCN1A* rs2298771 (G > A) vs. rs3812718 (C > T)			
GC	17 (8.42)	30 (13.4)	0.104
AC	58 (28.71)	63 (28.1)	0.89
GT	1 (0.5)	3 (1.3)	0.386
AT	126 (62.38)	128 (57.1)	0.228
*SCN1A* rs3812718 (C > T) vs. rs10188577 (A > G)			
CA	75 (37.13)	92 (41.1)	0.405
TA	93 (46.04)	97 (43.3)	0.57
CG	0	1 (0.4)	0.944
TG	34 (16.83)	34 (15.2)	0.64
*SCN1A* rs2298771 (G > A) vs. rs3812718 (C > T) vs. rs10188577 (A > G)			
GCA	17 (8.42)	30 (13.4)	0.1
ACA	58 (28.71)	63 (28.1)	0.89
GTA	0	2 (0.9)	-
ATA	93 (46.04)	95 (42.4)	0.397
GCG	0	0	0.94
ACG	0	1 (0.4)	-
GTG	1 (0.5)	1 (0.4)	0.94
ATG	33 (16.34)	33 (14.7)	-
*ABCB1* rs1128503 (T > C) vs. rs1045642 (T > C)			
TT	77 (38.12)	70 (31.3)	0.137
CT	6 (3.47)	5 (2.2)	0.446
TC	54 (26.73)	70 (31.3)	0.305
CC	65 (32.18)	79 (35.3)	0.5

**Table 6:** Logistic regression analysis of *SCN1A* rs2298771, *SCN1A* rs3812718 and clinical factors with drug response in epileptic children.

Predictors	Phenotype		
	OR	95% CI	*p*
(Intercept)	0.05	0.02–0.11	<0.001
*SCN1A* rs2298771 [GA]	2.43	0.99–6.50	0.062
Early onset	1.80	0.87–3.79	0.116
History of febrile seizure	3.23	1.42–7.87	0.007
History of infantile spasms	3.57	0.95–18.73	0.086
Intellectual disability	25.56	10.69–71.08	<0.001
Observations	211		
R^2^ Tjur	0.433		
(Intercept)	0.07	0.01–0.29	0.001
*SCN1A* rs3812718 [CT]	0.38	0.12–1.05	0.071
Early onset	2.30	0.88–6.13	0.091
History of febrile seizure	2.66	0.93–8.43	0.078
History of infantile spasms	3.11	0.63–22.05	0.202
Intellectual disability	34.16	10.27–162.22	<0.001
Observations	130		
R^2^ Tjur	0.482		

## Data Availability

The data used to support the findings of this study may be request from the corresponding author.

## Supplementary Materials

The following supporting information can be downloaded at: https://www.mdpi.com/article/10.3390/medicina60040637/s1, Table S1: Hardy-Weinberg equilibrium of genetic variants in study subjects.
