# Supplementary material for: Association between Genetic Polymorphism of SCN1A, GABRA1 and ABCB1 and Drug Responsiveness in Vietnamese Epileptic Children
Source: Medicina (Kaunas). 2024 Apr 16;60(4):637. doi: 10.3390/medicina60040637 (PMC11052159; doi:10.3390/medicina60040637)
Supplement: Supplementary file 1 [file medicina-60-00637-s001.zip › medicina-2924550-supplementary.pdf]

Supplementary data

Table S1

SNP	Genotype	Observed N = 213	Expected	χ2	p-HWE
SCN1A rs2298771	GG	2	3,052817	-	0,9411*
	AA	164	165,0528		
	AG	47	44,89437		
SCN1A rs3812718	CC	38	33,12676	1,955	0,3763
	CT	92	101,7465		
	TT	83	78,12676		
SCN1A rs10188577	AA	150	149,588	0,043	0,9786
	AG	57	57,82394		
	GG	6	5,588028		
GABRA1 rs2279020	GG	48	55,77934	4,551	0,1027
	GA	122	106,4413		
	AA	43	50,77934		
ABCB1 rs1128503	TT	88	86,19836	0,284	0,8674
	TC	95	98,60329		
	CC	30	28,19836		
ABCB1 rs1045642	TT	25	29,30047	1,595	0,4505
	TC	108	99,39906		
	CC	80	84,30047		

N: number of individuals, *Fisher's exact test
